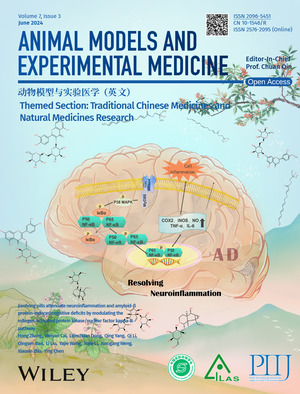# Cover Picture

**DOI:** 10.1002/ame2.12467

**Published:** 2024-07-07

## Abstract

The cover image is based on the article ‘Jiaohong pills attenuate neuroinflammation and amyloid‐β protein‐induced cognitive deficits by modulating the mitogen‐activated protein kinase/nuclear factor kappa‐B pathway’ (DOI 10.1002/ame2.12369) reported by Hong Zhang et al. Neuroinflammation is the primary cause of learning and memory impairment in Alzheimer's disease. Jiaohong pills, composed of Pericarpium Zanthoxyli and Radix Rehmanniae, can alleviate neuroinflammation in amyloid‐β protein‐induced‐Alzheimer's disease mice model by modulating the mitogen‐ activated protein kinase/nuclear factor kappa‐ B pathway signaling pathway, thereby further improving symptoms of learning and memory deficits.